# Identification of cell wall-associated kinases as important regulators involved in *Gossypium hirsutum* resistance to *Verticillium dahliae*

**DOI:** 10.1186/s12870-021-02992-w

**Published:** 2021-05-15

**Authors:** Jun Yang, Meixia Xie, Xingfen Wang, Guoning Wang, Yan Zhang, Zhikun Li, Zhiying Ma

**Affiliations:** State Key Laboratory of North China Crop Improvement and Regulation, Hebei Agricultural University, Baoding, 071001 China

**Keywords:** *Gossypium hirsutum*, WAK, *Verticillium dahliae*, Resistance, VIGS

## Abstract

**Background:**

Verticillium wilt, caused by the soil borne fungus *Verticillium dahliae*, is a major threat to cotton production worldwide. An increasing number of findings indicate that *WAK* genes participate in plant−pathogen interactions, but their roles in cotton resistance to *V. dahliae* remain largely unclear.

**Results:**

Here, we carried out a genome-wide analysis of WAK gene family in *Gossypium hirsutum* that resulted in the identification of 81 putative *GhWAKs*, which were all predicated to be localized on plasma membrane. In which, GhWAK77 as a representative was further located in tobacco epidermal cells using transient expression of fluorescent fusion proteins. All GhWAKs could be classified into seven groups according to their diverse protein domains, indicating that they might sense different outside signals to trigger intracellular signaling pathways that were response to various environmental stresses. A lot of *cis*-regulatory elements were predicted in the upstream region of *GhWAKs* and classified into four main groups including hormones, biotic, abiotic and light. As many as 28 *GhWAKs*, playing a potential role in the interaction between cotton and *V. dahliae*, were screened out by RNA-seq and qRT-PCR. To further study the function of *GhWAKs* in cotton resistance to *V. dahliae*, VIGS technology was used to silence *GhWAKs*. At 20 dpi, VIGSed plants exhibited more chlorosis and wilting than the control plants. The disease indices of VIGSed plants were also significantly higher than those of the control. Furthermore, silencing of *GhWAKs* significantly affected the expression of JA- and SA-related marker genes, increased the spread of *V. dahliae* in the cotton stems, dramatically compromised *V. dahliae*-induced accumulation of lignin, H_2_O_2_ and NO, but enhanced POD activity.

**Conclusion:**

Our study presents a comprehensive analysis on cotton WAK gene family for the first time. Expression analysis and VIGS assay provided direct evidences on *GhWAKs* participation in the cotton resistance to *V. dahliae*.

**Supplementary Information:**

The online version contains supplementary material available at 10.1186/s12870-021-02992-w.

## Background

Tetraploid *Gossypium hirsutum* is the most widely cultivated cotton species in the world and represents an important source of natural fiber and oilseed. Verticillium wilt, caused by the soil borne fungus *Verticillium dahliae*, is a major threat to cotton production [1]. Identification and characterization of genes associated with resistance is an important basis for potential understanding on the interaction between cotton and *V. dahliae*, which is necessary for the development of novel disease management methods and new varieties resistant to Verticillium wilt.

Plants live in a complex environment crowded with biotic stresses mainly caused by various phytopathogens and pests, and expose to abiotic stresses including cold, hot, drought and salinity. To overcome these stress challenges, plants have evolved a complex and efficient defense signaling network, which includes monitoring systems to perceive different stress-derived signals triggering specific defense responses [2]. Cell wall, a dynamic structure surrounding plant cell, has emerged as an essential monitoring system [3, 4]. Some receptor-like kinases (RLKs) have been identified as cell wall integrity sensors that are responsible for the communication between the cell wall and cytoplasm. Typically, RLKs contain a signal peptide (SP), transmembrane (TM) domain, and cytoplasmic kinase domain. They can be classified into more than 21 subfamilies according to their diverse extracellular domains [5]. Of which, wall-associated kinases (WAKs) are distinguished from the other RLKs by the presence of their unique extracellular epidermal growth factor (EGF)-like domains [5, 6].

In *Arabidopsis thaliana*, WAKs are encoded by 5 *WAKs* and 22 *WAKLs* (WAK-like genes) [7]*.* So far, *WAK* gene family was also identified in other plants, including *Oryza sativa* [8], *Brassica rapa* [9] and *Populus trichocarpa* [10]. It has been demonstrated that some WAKs are involved in plant development, abiotic and biotic stress responsiveness. Notably, most of *WAKs* were characterized from Arabidopsis and rice. Arabidopsis *AtWAK1*, the first identified WAK gene in plant, was shown to contribute to the immune response [11, 12]. A rice *WAK* gene, *OsDEES1* (DEFECT IN EARLY EMBRYO SAC1), played a role in the regulation of early embryo sac development [13]. *OsiWAK1* (*O. sativa indica* WAK-1) and *HvWAK1* (*Hordeum vulgare* WAK-1) were involved in plant root development [14, 15]. *Xa4*, encoding a WAK in rice, conferred race-specific durable resistance against *Xanthomonas oryzae* pv. *oryzae* by reinforcing the cell wall and increasing the production of jasmonate-isoleucine and phytoalexins [16]. *OsWAK1* (*O. sativa* WAK) and *OsWAK25* were up-regulated by wounding and salicylic acid (SA), and their overexpression led to higher resistance in transgenic rice lines against *Magnaporthe oryzae* [17, 18]*.* The other four rice *WAK* genes, including *OsWAK14*, *OsWAK91*, *OsWAK92* and *OsWAK112d*, were also suggested to be required for resistance to *M. oryzae* by loss-of-function mutants [19]. Beyond rice and Arabidopsis, *WAKs* have been characterized in response to pathogens as well in other plants, such as tomato *SlWAK1* (conferring resistance to *Pseudomonas syringae*) [20], maize *ZmWAK* (conferring resistance to *Sporisorium reilianum*) [21] and *ZmWAK-RLK1* (conferring resistance to *Setosphaeria turcica*) [22].

An increasing number of findings indicate that *WAK* genes participate in plant−pathogen interactions. Therefore, in our study, we used the latest *G. hirsutum* genome sequence data (HAU version 1.1 [23] to explore the *WAK* gene family, representing the first genome-wide identification of *GhWAKs*. Moreover, two *GhWAKs* were functionally characterized in response to *V. dahliae* infection using VIGS (virus induced gene silencing).

## Results

### *GhWAKs* identification and localization

**Fig. 1:**
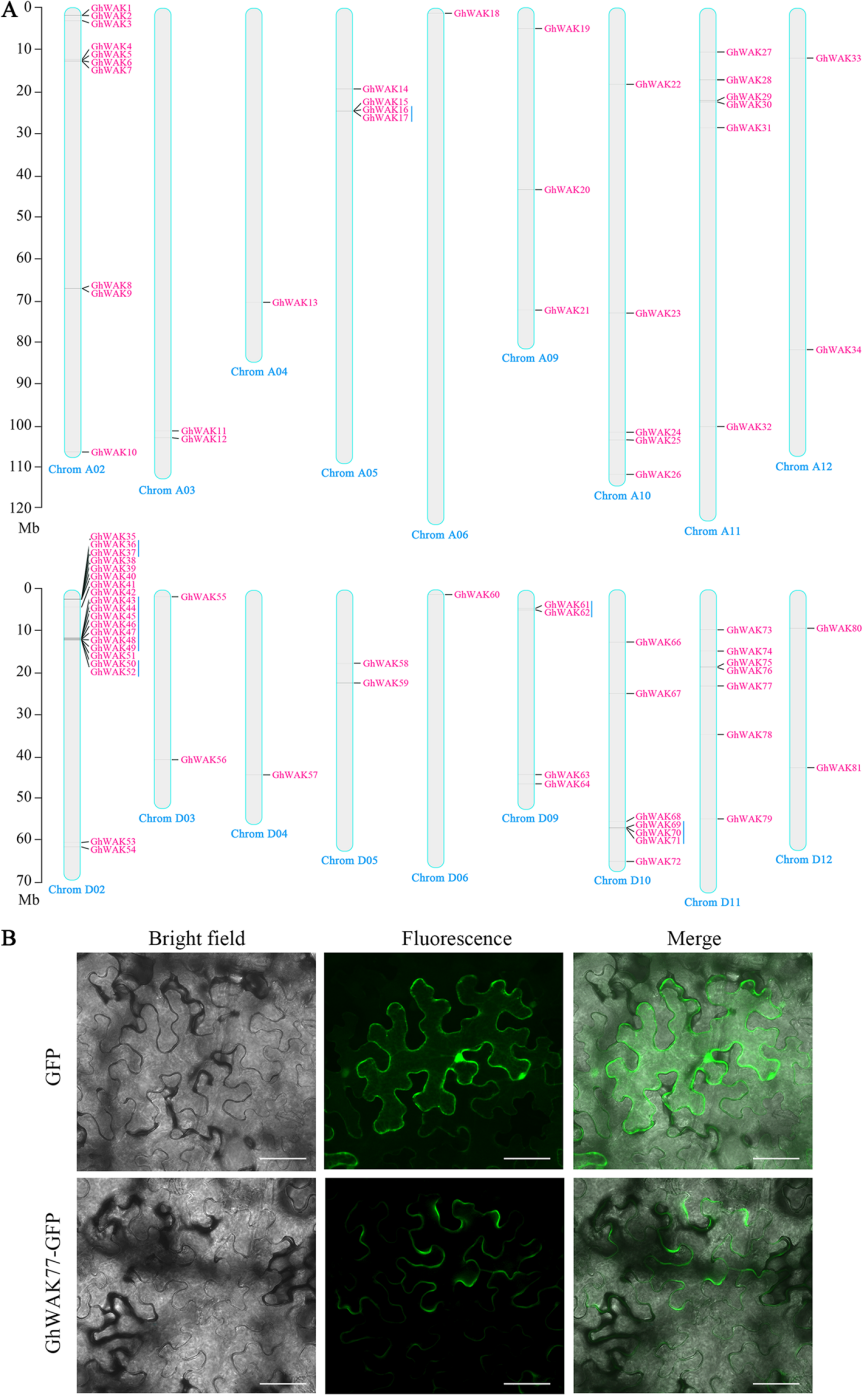
*WAKs* in *G. hirsutum*. **a**, Chromosomal distribution of *GhWAKs* in *G. hirsutum*. The chromosomal positions of *GhWAKs* were mapped according to the upland cotton genome using TBtools. The scale was in mega bases (Mb). The chromosome number was indicated at the bottom of each chromosome. Tandem duplicated genes were marked with blue lines. **b**, Subcellular localization of GhWAK77 in tobacco leaf epidermal cells. GFP (positive control) or GhWAK77 fused with the GFP (GhWAK77-GFP) proteins were transiently expressed in tobacco leaves via *A. tumefaciens* GV3101. At 48 h after agroinfiltration, GFP fluorescence was observed with confocal laser scanning microscope. Scale bars, 50 μm

In total, 81 *GhWAKs* as candidates were identified and named according to their chromosomal locations. These *GhWAKs* were marked on the physical map of 18 chromosomes (Fig. 1a) and one scaffold664 (*GhWAK65*). A total of 34 and 46 *GhWAKs* were distributed in the A and D sub-genomes, respectively. Chromosome D02 harbored the largest number of *GhWAKs* with 20 genes. Six pairs of tandem duplication events were found, including *GhWAK16/17*, *GhWAK36/37*, *GhWAK43/44–49*, *GhWAK50/52*, *GhWAK61/62* and *GhWAK69/70/71*. These results revealed that the evolution and expansion of *GhWAKs* happened in *G. hirsutum*, especially on chromosome D02. The detailed information about *GhWAKs*, including gene ID, open reading frame (ORF) length, amino acid length, protein molecular weight and isoelectric point, instability index and subcellular localization, was listed in Table 1.

**Table 1:** Detailed information of putative *G. hirsutum* WAK genes identified in this study

Gene Name	Gene ID	ORF (bp)	Length (aa)	MW (kDa)	pI	Instability index	Subcellular localization
*GhWAK1*	Ghir_A02G001840	2217	738	82.81	6.86	42.46	PM
*GhWAK2*	Ghir_A02G001850	2880	959	107.41	5.97	41.51	PM
*GhWAK3*	Ghir_A02G002660	2283	760	85.46	5.56	39.76	PM
*GhWAK4*	Ghir_A02G007280	2178	725	80.83	5.61	33.14	PM
*GhWAK5*	Ghir_A02G007310	2256	751	83.41	6.17	35.88	PM
*GhWAK6*	Ghir_A02G007330	2232	743	82.94	5.91	39.16	PM
*GhWAK7*	Ghir_A02G007350	2121	706	78.95	6.80	31.85	PM
*GhWAK8*	Ghir_A02G012070	2190	729	81.41	5.80	38.89	PM
*GhWAK9*	Ghir_A02G012080	2229	742	81.19	5.33	35.72	PM
*GhWAK10*	Ghir_A02G017660	2103	700	77.18	8.56	40.12	PM
*GhWAK11*	Ghir_A03G016250	1908	635	70.57	6.72	41.61	PM
*GhWAK12*	Ghir_A03G016560	2025	674	75.73	6.20	47.28	PM
*GhWAK13*	Ghir_A04G009230	1905	634	70.44	8.62	33.66	PM
*GhWAK14*	Ghir_A05G020230	2073	690	76.18	6.65	44.81	PM
*GhWAK15*	Ghir_A05G024460	2085	694	76.91	5.15	32.81	PM
*GhWAK16*	Ghir_A05G024500	2094	697	77.68	5.53	39.57	PM
*GhWAK17*	Ghir_A05G024510	2130	709	79.13	8.35	36.54	PM
*GhWAK18*	Ghir_A06G001260	2103	700	77.81	7.73	49.35	PM
*GhWAK19*	Ghir_A09G001860	2844	947	106.41	7.60	44.97	PM
*GhWAK20*	Ghir_A09G005720	1923	640	72.11	6.47	38.48	PM
*GhWAK21*	Ghir_A09G016250	1923	640	71.10	8.79	34.18	PM
*GhWAK22*	Ghir_A10G009180	2082	693	76.58	8.48	45.50	PM
*GhWAK23*	Ghir_A10G013470	2889	962	107.19	6.20	47.17	PM
*GhWAK24*	Ghir_A10G018760	2058	685	76.72	6.37	36.17	PM
*GhWAK25*	Ghir_A10G019250	2253	750	83.65	5.99	38.92	PM
*GhWAK26*	Ghir_A10G022760	1890	629	68.77	6.35	40.73	PM
*GhWAK27*	Ghir_A11G011010	1848	615	67.03	5.65	39.17	PM
*GhWAK28*	Ghir_A11G015050	2085	694	78.23	6.43	38.74	PM
*GhWAK29*	Ghir_A11G017400	1965	654	74.59	8.76	36.61	PM
*GhWAK30*	Ghir_A11G017530	2007	668	75.17	6.40	36.25	PM
*GhWAK31*	Ghir_A11G019930	2091	696	76.73	7.15	45.22	PM
*GhWAK32*	Ghir_A11G026030	1857	618	69.06	8.98	35.75	PM
*GhWAK33*	Ghir_A12G005550	1953	650	72.70	5.17	46.76	PM
*GhWAK34*	Ghir_A12G012670	1890	629	69.54	6.26	37.01	PM
*GhWAK35*	Ghir_D02G001920	2805	934	104.86	5.47	43.66	PM
*GhWAK36*	Ghir_D02G001930	2736	911	101.87	6.09	48.28	PM
*GhWAK37*	Ghir_D02G001940	2766	921	102.98	6.15	47.44	PM
*GhWAK38*	Ghir_D02G001960	2877	958	107.08	7.72	40.71	PM
*GhWAK39*	Ghir_D02G001970	3015	1004	112.34	7.20	43.96	PM
*GhWAK40*	Ghir_D02G001980	2853	950	105.62	5.97	40.01	PM
*GhWAK41*	Ghir_D02G003070	2925	974	109.39	5.69	39.50	PM
*GhWAK42*	Ghir_D02G007710	1929	642	71.21	6.81	39.67	PM
*GhWAK43*	Ghir_D02G007720	2052	683	75.70	5.31	38.92	PM
*GhWAK44*	Ghir_D02G007730	2238	745	82.72	5.20	40.76	PM
*GhWAK45*	Ghir_D02G007740	2253	750	84.49	6.17	38.33	PM
*GhWAK46*	Ghir_D02G007750	2196	731	81.89	5.98	37.64	PM
*GhWAK47*	Ghir_D02G007760	2049	682	75.75	5.36	36.84	PM
*GhWAK48*	Ghir_D02G007780	2313	770	85.69	6.12	36.93	PM
*GhWAK49*	Ghir_D02G007790	2214	737	81.56	5.98	37.05	PM
*GhWAK50*	Ghir_D02G007800	1905	634	71.16	5.77	33.20	PM
*GhWAK51*	Ghir_D02G007810	2163	720	80.85	6.13	41.17	PM
*GhWAK52*	Ghir_D02G007820	2151	716	80.23	6.54	35.66	PM
*GhWAK53*	Ghir_D02G017510	1908	635	70.61	6.55	44.29	PM
*GhWAK54*	Ghir_D02G017820	1890	629	70.82	6.12	48.70	Ex, PM
*GhWAK55*	Ghir_D03G001900	2100	699	76.95	8.54	38.15	PM
*GhWAK56*	Ghir_D03G011850	2076	691	76.83	8.55	33.01	PM
*GhWAK57*	Ghir_D04G013370	1920	639	71.07	8.58	32.21	PM
*GhWAK58*	Ghir_D05G020210	2181	726	80.64	7.19	45.94	PM
*GhWAK59*	Ghir_D05G024300	3069	1022	114.00	6.47	33.32	PM
*GhWAK60*	Ghir_D06G001130	2103	700	77.76	7.74	47.04	PM
*GhWAK61*	Ghir_D09G001670	2862	953	106.48	6.04	42.26	PM
*GhWAK62*	Ghir_D09G001690	2850	949	106.27	5.71	43.80	PM
*GhWAK63*	Ghir_D09G015720	1914	637	70.65	8.74	35.45	PM
*GhWAK64*	Ghir_D09G018010	1995	664	75.49	6.23	42.60	PM
*GhWAK65*	Ghir_D09G025850	1971	656	74.68	6.20	43.00	PM
*GhWAK66*	Ghir_D10G010060	2082	693	76.31	8.53	46.47	PM
*GhWAK67*	Ghir_D10G014200	2898	965	107.43	5.62	47.03	PM
*GhWAK68*	Ghir_D10G020270	2049	682	76.45	6.53	35.60	PM
*GhWAK69*	Ghir_D10G020870	2091	696	77.38	6.38	34.71	PM
*GhWAK70*	Ghir_D10G020880	2310	769	86.29	6.24	34.86	PM
*GhWAK71*	Ghir_D10G020930	2307	768	86.09	5.67	35.08	PM
*GhWAK72*	Ghir_D10G025210	1830	609	66.92	6.20	45.83	PM
*GhWAK73*	Ghir_D11G010940	1923	640	69.64	5.89	40.12	PM
*GhWAK74*	Ghir_D11G015120	1902	633	71.17	6.12	44.54	PM
*GhWAK75*	Ghir_D11G017450	1992	663	74.61	8.03	50.47	PM
*GhWAK76*	Ghir_D11G017550	2058	685	75.91	5.97	47.03	PM
*GhWAK77*	Ghir_D11G020010	2094	697	76.74	6.92	46.71	PM
*GhWAK78*	Ghir_D11G023010	2040	679	75.99	6.72	36.11	PM
*GhWAK79*	Ghir_D11G026200	1908	635	70.99	8.78	38.79	PM
*GhWAK80*	Ghir_D12G005550	2004	667	74.32	5.50	46.69	PM
*GhWAK81*	Ghir_D12G012920	1896	631	69.95	6.55	36.83	PM

*PM* plasma membrane, *Ex* extracellular

All GhWAKs were predicated to be localized on plasma membrane (PM) (Table 1). In which, GhWAK77 as a representative was further located in tobacco epidermal cells using transient expression of fluorescent fusion proteins. The images clearly showed that fluorescent signal corresponding to the sole *gfp* (green fluorescent protein) gene was observed in PM, cytoplasm and nucleus. However, the fluorescent signal corresponding to *GhWAK77*-*gfp* was solely shown in PM (Fig. 1b). These suggested that GhWAKs might be a potential connector responsible for communication between inside and outside of the cell.

### GhWAKs have conservative kinase domains and diverse extracellular domains

**Fig. 2:**
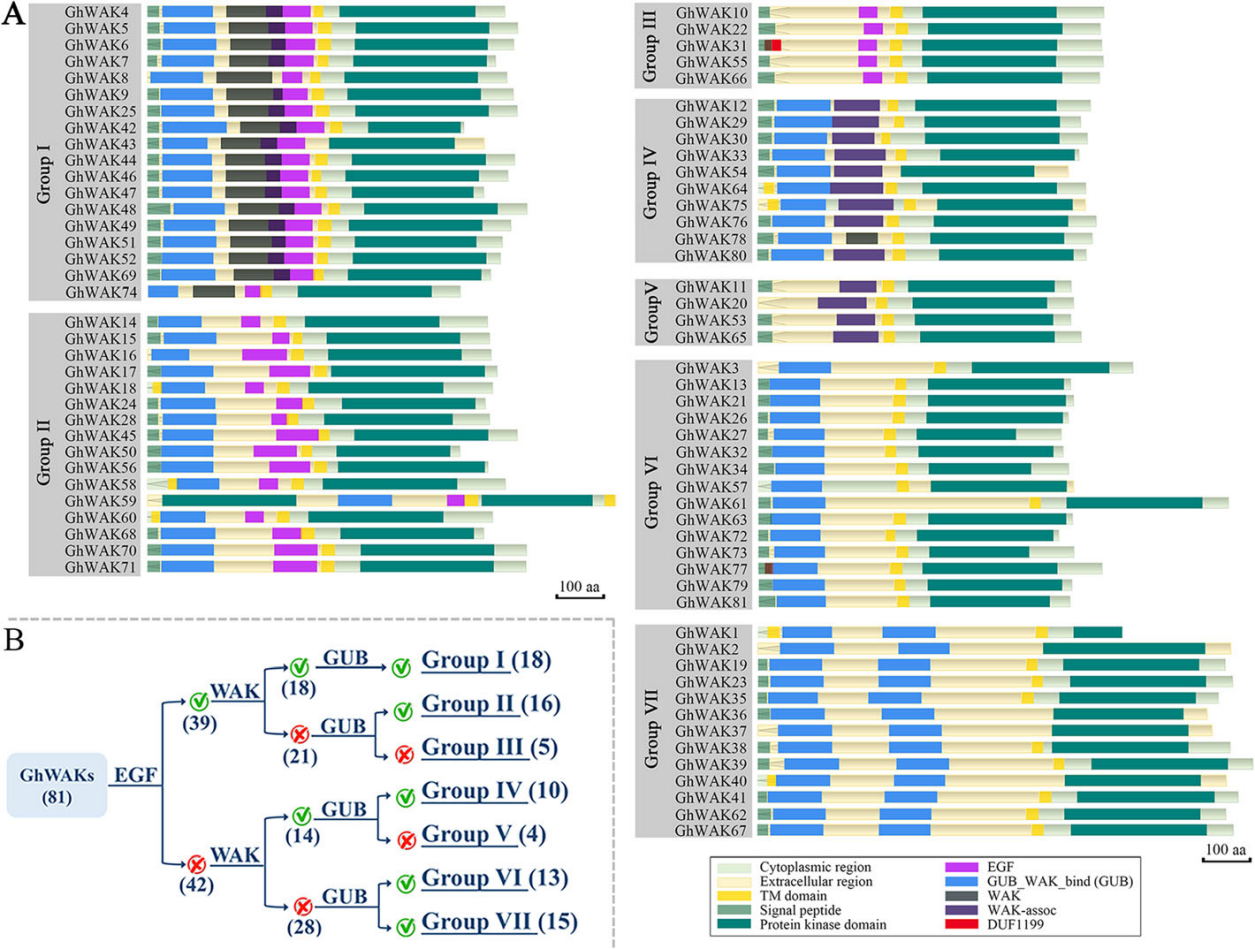
Protein domain analyses of GhWAKs. **a**, Domain organization of GhWAKs. **b**, Grouping of GhWAKs. Based on the presence (green checkmarks) or absence (red crosses) of some domains, GhWAKs were divided into four groups. The numbers in brackets represent the number of GhWAKs

The majority of *GhWAKs* have 3–4 introns and show similar exon-intron structure (Figure S1). A total of six conserved protein domains were identified in GhWAKs, including GUB_WAK_bind (wall-associated receptor kinase galacturonan-binding, PF13947), WAK (wall-associated kinase, PF08488), WAK assoc. (wall-associated receptor kinase C-terminal, PF14380), EGF (EGF, PF00008; cEGF, PF12662; hEGF, PF12661; EGF_CA, PF07645; EGF_3, PF12947), DUF1199 (domain of unknown function, PF06712) and protein kinase domain (pkinase, PF00069; pkinase_Tyr, PF07714; kinase-like, PF14531; protein-kinase domain of FAM69, PF12260) (Fig. 2a). Cytoplasmic, extracellular and TM regions were predicated in the majority of GhWAKs, further indicating that they were PM proteins. Typical *WAK* encodes a transmembrane protein with a cytoplasmic kinase domain and an extracellular region. However, several proteins showed uncommon structural characteristics, such as the kinase domain in extracellular region, double TMs and kinase domains. All GhWAKs were classified into seven groups according to their protein domain analysis (Fig. 2b). The members in Group I, Group II and Group III were typical WAKs that contain EGF domain in extracellular region. The other four Groups, including IV, V, VI and VII, do not contain EGF. GhWAKs in Group I and IV contain both WAK and GUB domain. Inversely, GhWAKs in Group III neither contain WAK nor GUB domain. GhWAKs in Group II, VI and VII only contain GUB domain. However, II and VI are one-GUB-domain groups, and VII are two-GUB-domain group. GhWAKs in Group V only contain WAK domain. Additionally, DUF1199 domain was found in GhWAK31 and GhWAK77. Different types and numbers of extracellular domains were present in GhWAKs, indicating that they might sense or bind different outside signaling to trigger intracellular signaling pathways that control plant development and response to various environmental stresses.

### Prediction of putative *cis*-regulatory elements in *GhWAK* promoters

**Fig. 3:**
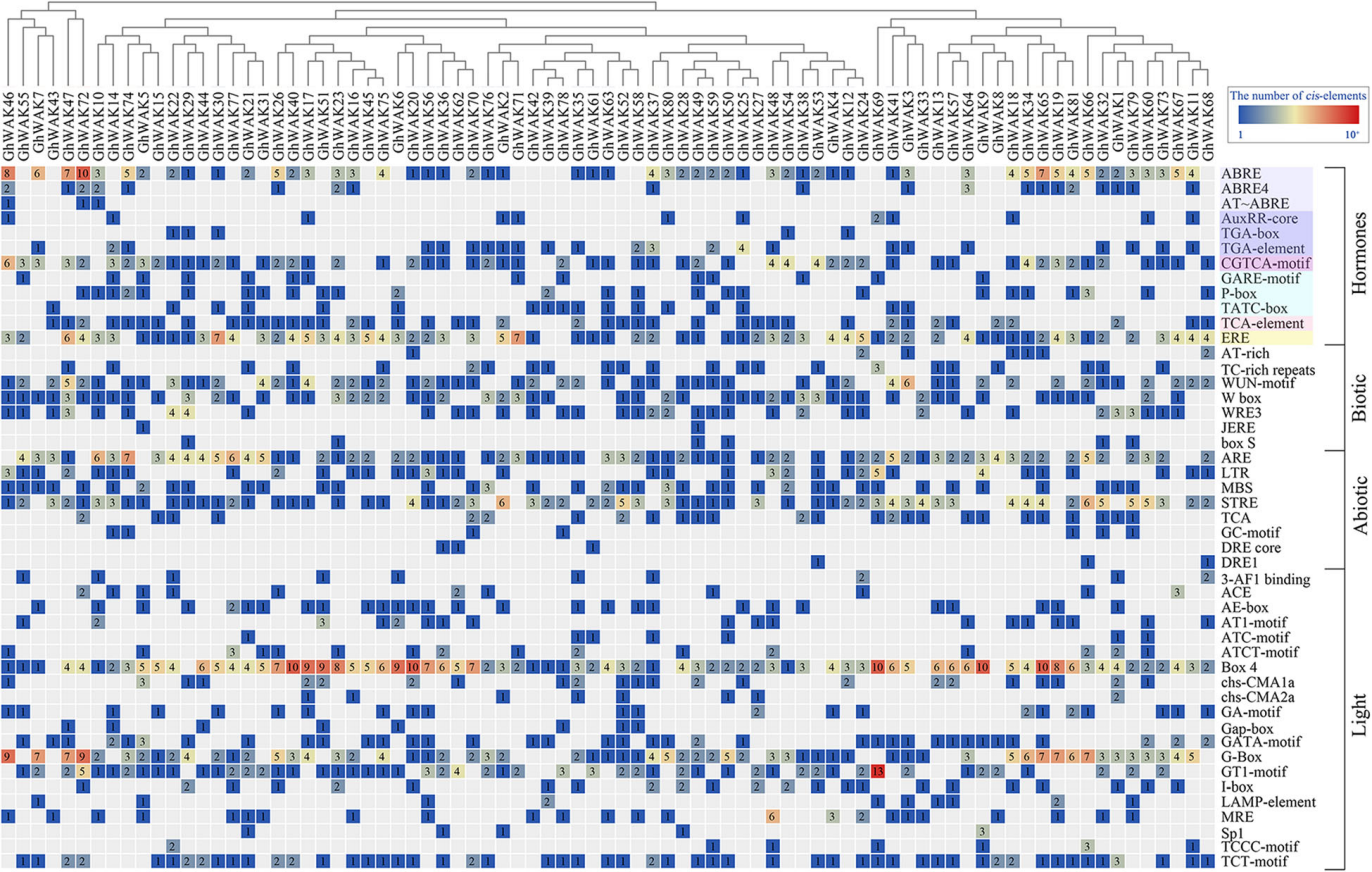
Potential *cis*-elements in a 2 kb 5′ flanking region upstream from the start codon of each *GhWAK*. The number of each *cis*-element was shown, and the back-color changes from blue to red as the number increase. All *cis*-regulatory elements were classified into four groups, including hormones, biotic, abiotic and light

The 2-kb region upstream of the translation start site of all *GhWAKs* were considered the promoter and analyzed for the potential roles of *cis*-regulatory elements (Fig. 3). These *cis*-regulatory elements were classified into four main groups including hormones, biotic, abiotic and light. Twelve hormone-responsive regulatory elements associated with abscisic acid (ABA) (ABRE, ABRE4 and AT-ABRE), auxin (IAA) (AuxRR-core, TGA-box and TGA-element), methyl jasmonate (MeJA) (CGTCA-motif), gibberellin (GA) (GARE-motif, P-box and TATC-box), SA (TCA-element) and ethylene (ET) (ERE), were identified. Of which, ABRE-motif, CGTCA-motif and ERE were enriched in the most of *GhWAK* promoters, indicating that they might be widely induced by ABA, JA and ET. The biotic stress-related regulatory elements, such as AT-rich, TC-rich repeats, W-box, WUN-motif, WRE3, JERE and box S, were involved in elicitor-mediated activation, wounding and pathogen responsiveness. In addition, eight abiotic-responsive regulatory elements, associated with anaerobic induction (ARE and GC-motif), low-temperature responsiveness (LTR), drought-inducibility (MBS, DRE core and DRE1), heat shock, osmotic stress, low pH, nutrient starvation (STRE) and stress-related (TCA), were identified in the *GhWAK* promoter regions. Moreover, various light-responsive elements were present in the promoters of *GhWAKs*. Especially, Box 4 and G-Box were widely harbored. These results indicated that *GhWAKs* might play vital roles in the response to various stresses, hormones and light.

### *GhWAKs* were significantly induced by *V. dahliae* infection

**Fig. 4:**
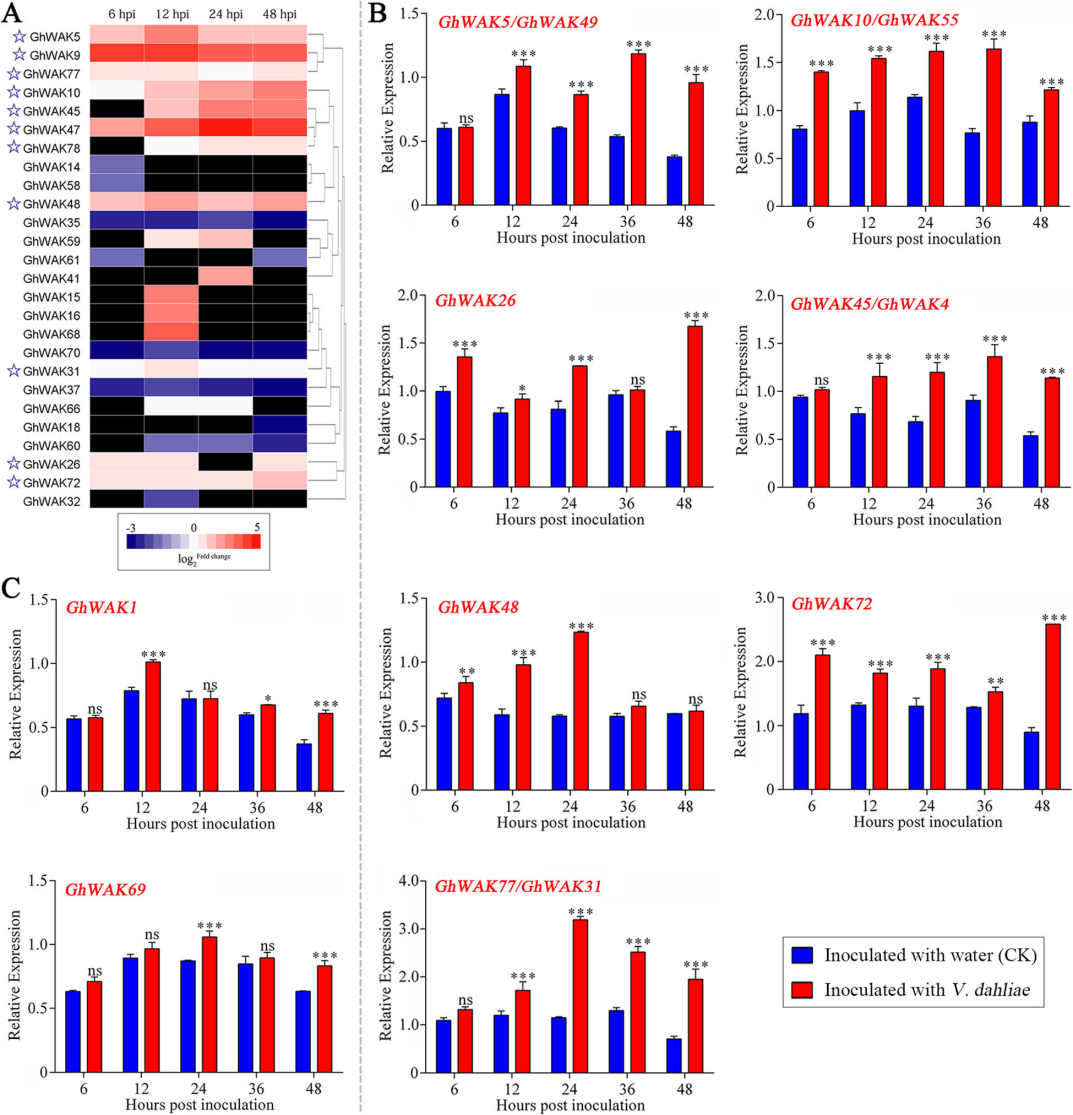
Expression of *GhWAKs* in response to *V. dahliae* infection. **a**, Heatmap representation for the expression patterns of 26 *GhWAKs* differentially expressed as a result of cotton inoculation with *V. dahliae*, compared to the respective control. Expression levels of genes were shown as the Log_2_ Fold change (FC). FC is a ratio of treatment FPKM to control FPKM that obtained from the RNA-Seq data. Higher and lower transcriptional level are indicated by pink and blue, respectively, and no detected expression is indicated by dark color. **b** and **c**, The expression analysis of *GhWAKs* from *G. hirsutum* ND601 induced with *V. dahliae* by qRT-PCR. The relative gene expression level was calculated using the comparative 2^-*ΔΔCt*^ method with *GhHis3* as internal control. The bar represents the standard error (SE) calculated from three independent experiments. Asterisks indicate statistically significant differences according to Sidak’s multiple comparisons test (**P < 0.001, ***P < 0.01; ns, no significant)

To identify *GhWAKs* that were related to *V. dahliae* infection, two-fold changes were applied in transcript expression profiles from RNA-seq as minimum cutoffs. As a result, 26 *GhWAKs* were screened out, including 17 up-regulated and 9 down-regulated genes (Fig. 4a). Of which, 11 *GhWAKs*, including *GhWAK5*, *GhWAK9*, *GhWAK77*, *GhWAK10*, *GhWAK45*, *GhWAK47*, *GhWAK78*, *GhWAK48*, *GhWAK31*, *GhWAK26* and *GhWAK72*, were significantly up-regulated in at least three time points, suggesting that they continuously responded to *V. dahliae* infection. Their expression profiles were further verified through real-time quantitative reverse transcription PCR (qRT-PCR). The expression results of them in response to the *V. dahliae* infection from qRT-PCR were consistent with those found in RNA-seq data (Fig. 4b)*.* Due to the high degree of sequence similarity in *GhWAKs* family, it was difficult to design specific primers for four gene pairs, including *GhWAK4*/*GhWAK45*, *GhWAK5*/*GhWAK*49, *GhWAK10/GhWAK55*, and *GhWAK31*/*GhWAK77.* The results of qRT-PCR indicated that these four pairs of *GhWAKs* were dramatically up-regulated. According to RNA-seq data, *GhWAK4*, *GhWAK49* and *GhWAK55* did not show to be up-regulated. Thus, the expression changes found using qRT-PCR probably more represent the responses of *GhWAK45*, *GhWAK5* and *GhWAK10* to *V. dahliae* infection. In addition, the other 45 *GhWAKs* that did not show differential expression in RNA-seq data were further detected through qRT-PCR. As a result, *GhWAK1* and *GhWAK69* showing higher transcription levels in cotton seedlings inoculated with *V. dahliae* than that in control was screened out complementally (Fig. 4c). Finally, a total of 28 *GhWAKs* were found to play a potential role in the interaction between cotton and *V. dahliae*.

### Silencing *GhWAKs* compromised cotton resistance to Verticillium wilt

**Fig. 5:**
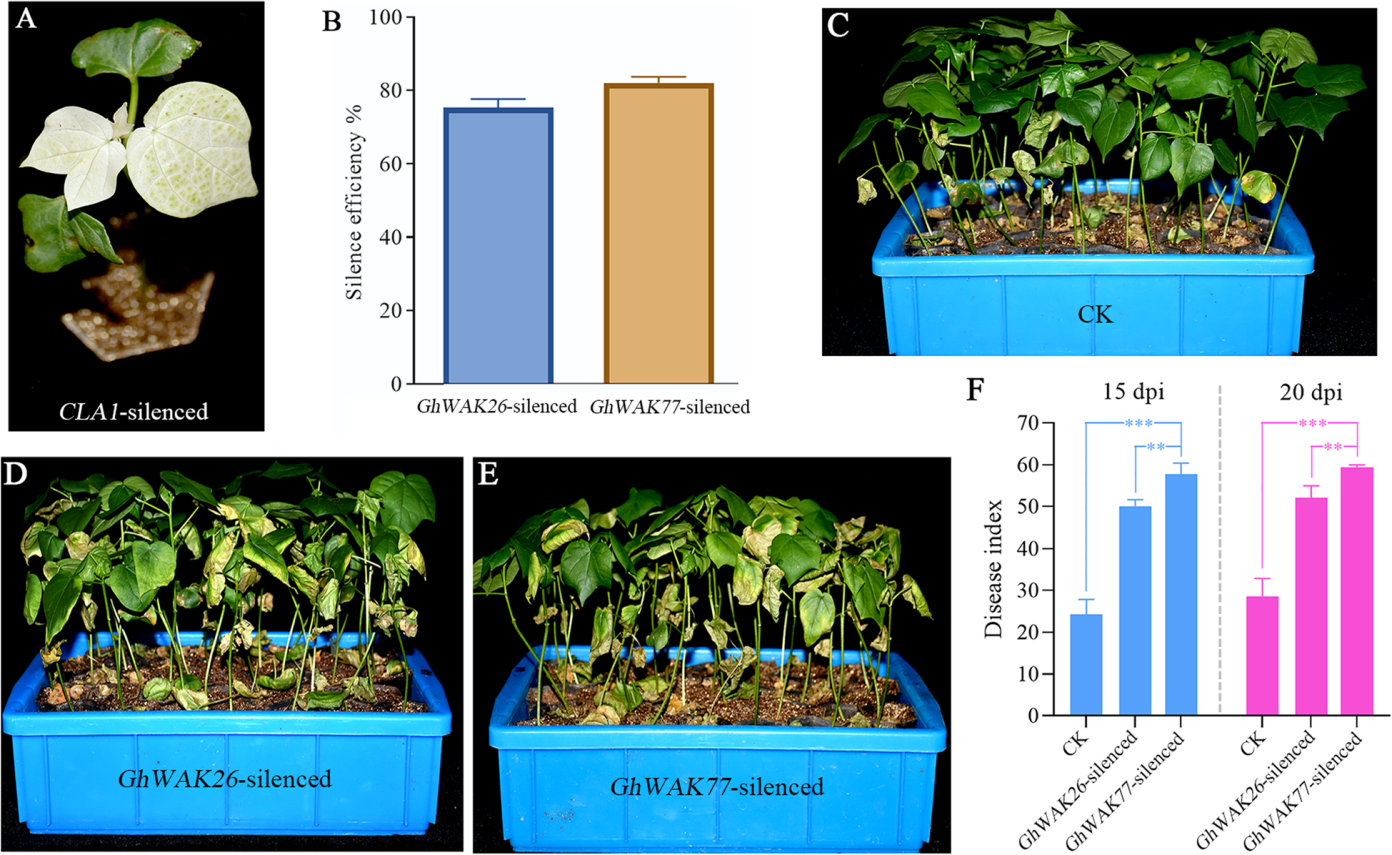
Silencing of *GhWAKs* in cotton compromised plant resistance to *V. dahliae*. **a**, Albinotic *CLA1*-silenced seedling served as the indicator of successful VIGS. **b**, VIGS reduced the expression of *GhWAKs* by about 80%. **c**, Disease symptom for control at 15 dpi. **d**, Disease symptom for *GhWAK26*-silenced plants at 15 dpi. **e**, Disease symptom for *GhWAK77*-silenced plants at 15 dpi. **f**, Disease indices of *GhWAK26*- and *GhWAK77*-silenced plants at 15 dpi and 20 dpi. The results were evaluated by three replications, and each contained at least 30 plants. Asterisks indicate statistically significant differences according to Sidak’s multiple comparisons test (*P < 0.05; **P < 0.01; ***P < 0.001; ns, no significant)

*GhWAK26* and *GhWAK77* showed obviously and persistently up-regulated expression to the infection from *V. dahliae* (Fig. 4)*.* In addition, they contain *cis*-elements in their promoters associated with MeJA and SA, which play key roles in cotton resistance to *V. dahliae*. Thus, to further reveal the function of *GhWAKs* in cotton resistance to *V. dahliae*, *GhWAK26* and *GhWAK77* were prioritized for study as representatives here using tobacco rattle virus (TRV) based VIGS system. At approximately two weeks post-infiltration with a mixture of Agrobacterium cultures containing pTRV1 and pTRV2-*CLA1*, a strong photobleaching phenotype was shown on the newly emerging true leaves (Fig. 5a), indicating that VIGS system worked well. Then, the expression of *GhWAK26* and *GhWAK77* was detected in the leaves infiltrated with pTRV2-*GhWAK26* and pTRV2-*GhWAK77*, respectively. As shown in Fig. 5b, the expression of *GhWAK26* and *GhWAK77* was reduced by about 80%, suggesting VIGS triggered their silencing in cotton plants. At 20 days post inoculation (dpi), VIGSed plants (Fig. 5d and e) exhibited more chlorosis and wilting than the control plants infiltrated with Agrobacterium cultures containing empty vector pTRV1 and pTRV2 (Fig. 5c). The disease indices of VIGSed plants were also significantly higher than those of the control at 15 dpi and 20 dpi (Fig. 5f). Therefore, the results of VIGS assays suggested that *GhWAK26* and *GhWAK77* were important participants in cotton resistance to *V. dahliae* infection.

### Silencing *GhWAKs* increased the spread of *V. dahliae* in cotton stems

**Fig. 6:**
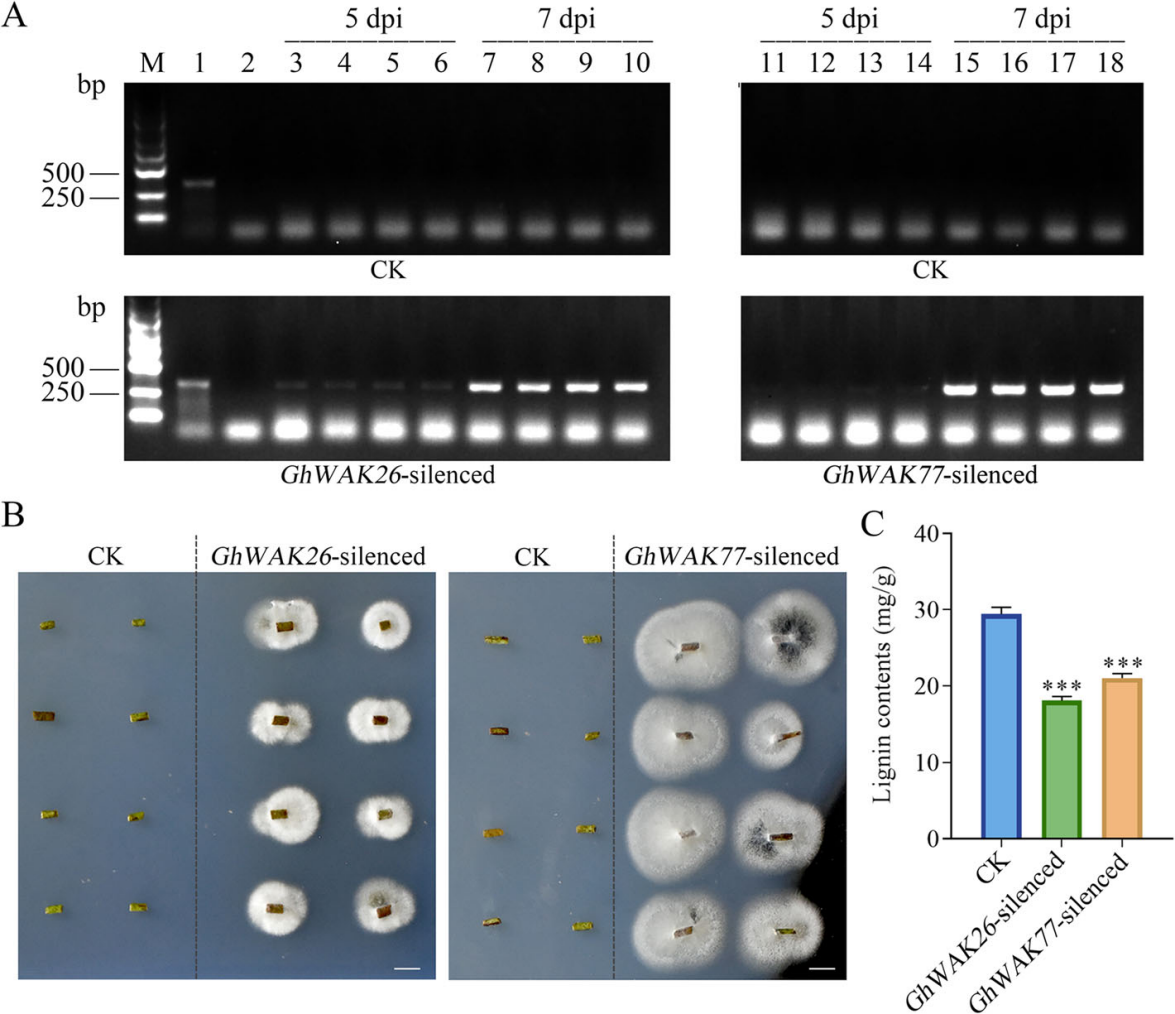
Silencing of *GhWAKs* increased the spread of *V. dahliae* in the cotton stems. **a**, Detection for *V. dahliae* in cotton stems at 5 dpi and 7 dpi by PCR. M, marker. DNA templates for PCR were extracted from *V. dahliae* spores as positive control (lane 1), water as negative control (lane 2), and cotton seedling stems (lane 3–18). **b**, Isolation of *V. dahliae* from the stems of *GhWAK26*-silenced and *GhWAK77*-silenced cotton plants by PDA cultivation. Bars = 0.5 cm. **c**, The lignin content in *GhWAK*-silenced plants and CK. The results from three biological replicates are shown with mean ± SE. Asterisks represent *P* values (***P < 0.001; Dunnett’s multiple comparisons test)

After inoculation, *V. dahliae* in cotton stem was detected by PCR. No specific amplification products from *V. dahliae* were shown in CK at 5 dpi and 7 dpi, indicating that *V. dahliae* had not yet invaded the stems or multiplied in large quantities (Fig. 6a and Figure S2). However, at 5 dpi, few specific products from *V. dahliae* were amplified in *GhWAK26*-silenced and *GhWAK77*-silenced plants, representing a small amount of pathogen invasion. Further, at 7 dpi, the bright bands amplified from *GhWAK26*-silenced and *GhWAK77*-silenced plant stems appeared on agarose gels, indicating that *V. dahliae* had invaded largely. In addition, pathogen isolation on potato dextrose agar (PDA) showed that a large number of *V. dahliae* grew out from the stems of *GhWAK26*-silenced and *GhWAK77*-silenced cotton plants, while no mycelium was shown from the control (Fig. 6b). Both PCR detection and PDA culture results suggested that silencing *GhWAKs* significantly increased the spread of *V. dahliae* in the cotton stems.

Lignin is considered to play an important role in preventing cotton from the infection of *V. dahliae*. Therefore, we further compared the changes of lignin content in *GhWAK*-silenced cotton stems with CK. The results showed that the lignin content in *GhWAK*-silenced plants was significantly lower than that in CK (Fig. 6c), which might affect the stem structure and then reduce the prevention of cotton from *V. dahliae* infection.

### Silencing *GhWAKs* dramatically affected *V. dahliae*-induced H_2_O_2_, nitric oxide (NO) and peroxidase (POD)

**Fig. 7:**
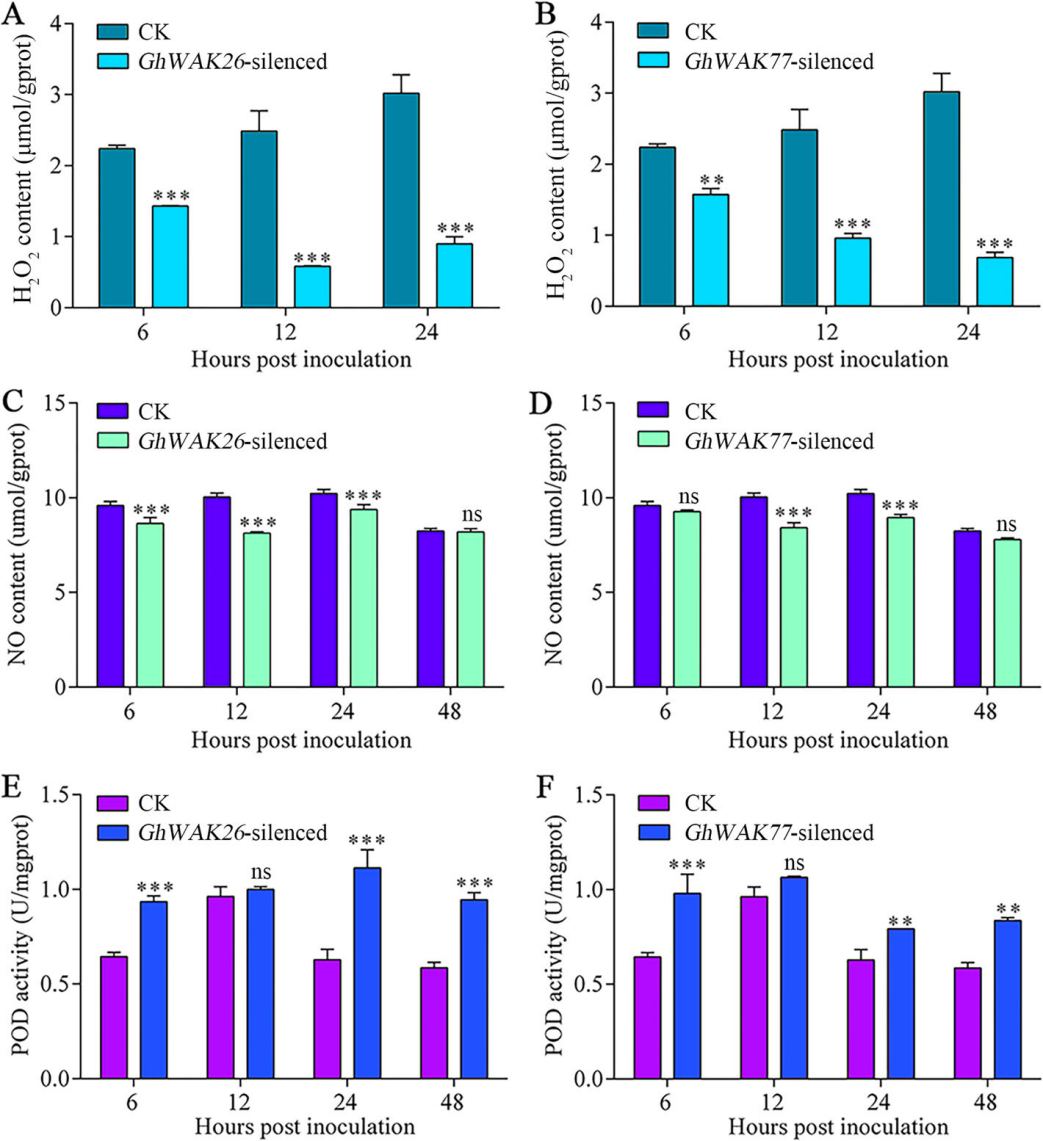
Silencing of *GhWAKs* dramatically compromised *V. dahliae*-induced accumulation of H_2_O_2_ (**a** and **b**) and NO (**c** and **d**), but enhanced POD activity (**e** and **f**). The results from three biological replicates are shown with mean ± SE. Asterisks represent *P* values (**P < 0.001, ***P < 0.001; ns, no significant; Sidak’s multiple comparisons test)

The content of H_2_O_2_ and NO, and POD activity in *GhWAK*-silenced plants inoculated with *V. dahliae* were further measured. *GhWAKs* silencing caused lower levels of H_2_O_2_ at 6 h post inoculation (hpi), 12 hpi and 24 hpi (Fig. 7a and b). Both *GhWAK26*- and *GhWAK77*-silenced plants accumulated greatly depressed levels of NO comparing with CK (Fig. 7c and d). However, the activity of POD significantly elevated in *GhWAK26*- and *GhWAK77*-silenced plants at 6 hpi, 24 hpi and 48 hpi, except at 12 hpi (Fig. 7e and f).

### Silencing *GhWAKs* significantly affected the expression of JA and SA-related marker genes

**Fig. 8:**
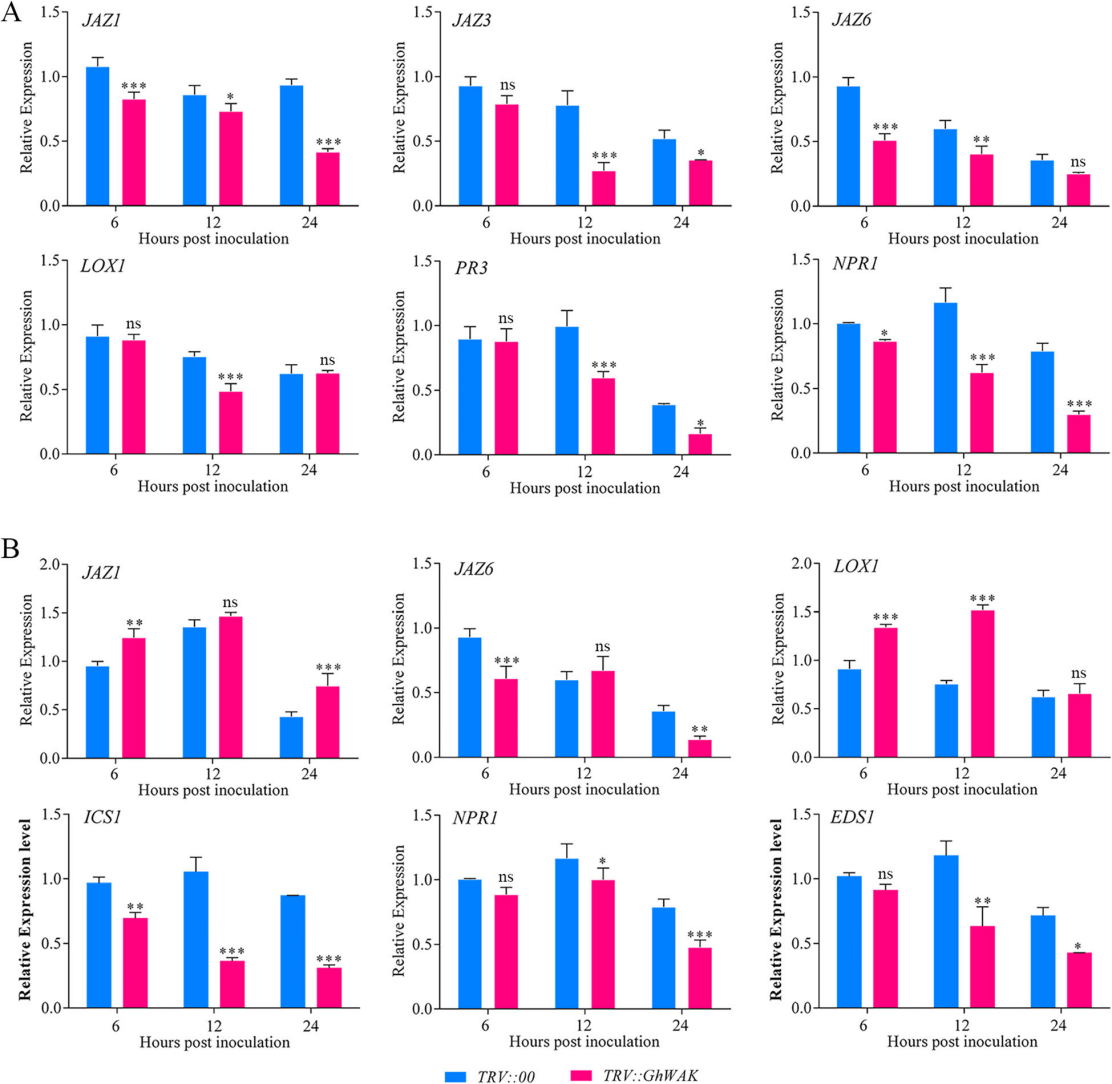
Silencing of *GhWAKs* affected the expression of marker genes in JA and SA signaling pathways. **a**, The expression level of six marker genes in *GhWAK26*-silenced plants inoculated with *V. dahliae*. **b**, The expression level of six marker genes in *GhWAK77*-silenced plants inoculated with *V. dahliae*. *JAZs*, *LOX1* and *PR3* are the marker genes involved in JA signaling pathway. *ICS1*, *NPR1* and *EDS1* are the marker genes involved in SA signaling pathway. The results from three biological replicates are shown with mean ± SE. Asterisks represent *P* values (*P < 0.05; **P < 0.01; ***P < 0.001; ns, no significant; Sidak’s multiple comparisons test)

Further, the expression of several JA and SA-related marker genes involved in plant defense signaling pathways was detected. The expression of *JAZ1* (jasmonate-zim-domain protein), *JAZ3*, *JAZ6*, *LOX1* (lipoxygenase) (JA-related marker genes), *PR3* (pathogenesis related protein) and *NPR1* (nonexpresser of PR protein) (SA-related marker genes) were significantly down-regulated after silencing *GhWAK26* in cotton (Fig. 8a). In *GhWAK77*-silenced plants, *JAZ6* and three important genes involved in the SA signaling pathway, including *ICS1* (isochorismate synthase), *NPR1* and *EDS1* (enhanced disease susceptibility), were down-regulated comparing with control. On the contrary, the expression of *JAZ1* and *LOX1* were significantly up-regulated due to the silencing of *GhWAK77* (Fig. 8b). These results indicated that *GhWAK26 and GhWAK77* might involve in cotton resistance to *V. dahliae* through SA and JA signaling pathways.

## Discussion

WAK gene family has been analyzed in several plant species, such as *A. thaliana* [7], *O. sativa* [8], *P. trichocarpa* [10] and *B. rapa* [9]. Some *WAKs* have been implicated in the response to pathogen infection. Examples are Arabidopsis *Wak1* [12], maize *ZmWAK-RLK1* (*Htn1*) and *ZmWAK* (*qHSR1*) [21, 22], wheat *Stb6* and TaWAK6 [24, 25], rice *Xa4*, *OsWAK1* and *OsWAK91* [16, 17, 26], and orange *CsWAKL08* [27], conferring host plant disease resistance. In the present work, a total of 81 *GhWAKs* were systematically identified and analyzed for the first time from a high-quality *G. hirsutum* genome (Table 1) [23]. Of which, 28 *GhWAKs* were potentially involved into the interaction between cotton and *V. dahliae* (Fig. 4). Especially, silencing of *GhWAK26* or *GhWAK77* dramatically reduced the resistance of cotton plants to *V. dahliae* infection (Fig. 5), suggesting that *WAKs* were important resistance genes during cotton–pathogen interactions.

At the PM, RLKs as cell-surface receptors can perceive and process extracellular danger signals to trigger plant defense responses [28]. WAK belongs to RLK subfamily. All GhWAKs contain a typical eukaryotic kinase domain that is mostly present in intracellular region and relatively well conserved (Fig. 2a). In addition, GhWAKs locate on PMs in all probability (Table 1, Fig. 1), suggesting that GhWAKs have potential roles in communicating between inside and outside of the cell. In order to penetrate plant roots to gain access to the xylem and to spread in the vascular system, *V. dahliae* usually secretes various toxins and carbohydrate active enzymes, including glycoproteins and cell wall-degrading enzymes [29, 30]. Therefore, it is conceivable that *V. dahliae* infection affects plant cell wall integrity (CWI) and generates some degradation products, which are important defense signals [31]. In the extracellular region, GhWAKs contain five different domains (Fig. 2a), which may sense CWI or interact with different components of these extracellular matrix, such as glycine-rich protein, pectin and oligogalacturonides (OGs) [32–34].

At present, the molecular mechanism of *WAK*-mediated resistance remains largely unknown. However, some defence responses associated with WAKs have been reported, including cell wall reinforcement [16], pathogenesis-related genes activation [18], SA or JA accumulation [27], POD and superoxide dismutase activities [27], and reactive oxygen species (ROS) homeostasis [27]. Here, silencing *GhWAKs* resulted in the up- or down-regulation of several genes (Fig. 8) and depressed cotton resistance to *V. dahliae*. Among them, *JAZ* and *LOX* are associated with JA-mediated defense responses [35]. *NPR1, ICS1* and *EDS1* are associated with SA-mediated defense responses [36]. The two phytohormones, JA and SA, have been known to be involved into the regulation of plant resistance against *V. dahliae* [37, 38]*.* In addition, some hormone-responsive and biotic stress-related regulatory elements were enriched in the promoters of *GhWAKs* (Fig. 3). Thus, these findings suggest that *GhWAK* function as a mediator to active intracellular SA and JA signaling pathways to regulate cotton resistance.

*V. dahliae* is a vascular pathogen that penetrates the host roots and then extends to other overground parts of plant through the process of transpiration [29, 37]. The improvement of physical, chemical and structural barriers, such as ROS, NO, cell wall, lignin, callose and POD, contributes to preventing expansion and reducing colonization of *V. dahliae* in cotton tissues [37, 39–41]. In this study, more *V. dahliae* was detected in *GhWAK26*-silenced or *GhWAK77*-silenced plants with lower lignin contents than in CK (Fig. 6). Moreover, silencing of *GhWAKs* in cotton plants dramatically compromised *V. dahliae*-induced accumulation of H_2_O_2_ and NO, but enhanced POD activity (Fig. 7). These findings demonstrate that *GhWAKs* play roles in preventing pathogen spreading at least in part by regulating the accumulation of lignin, H_2_O_2_ and NO, and the activity of POD. Overall, these results augment our knowledge about cotton WAK gene family, and particularly promote the understanding on their function in disease resistance.

## Conclusions

**Fig. 9:**
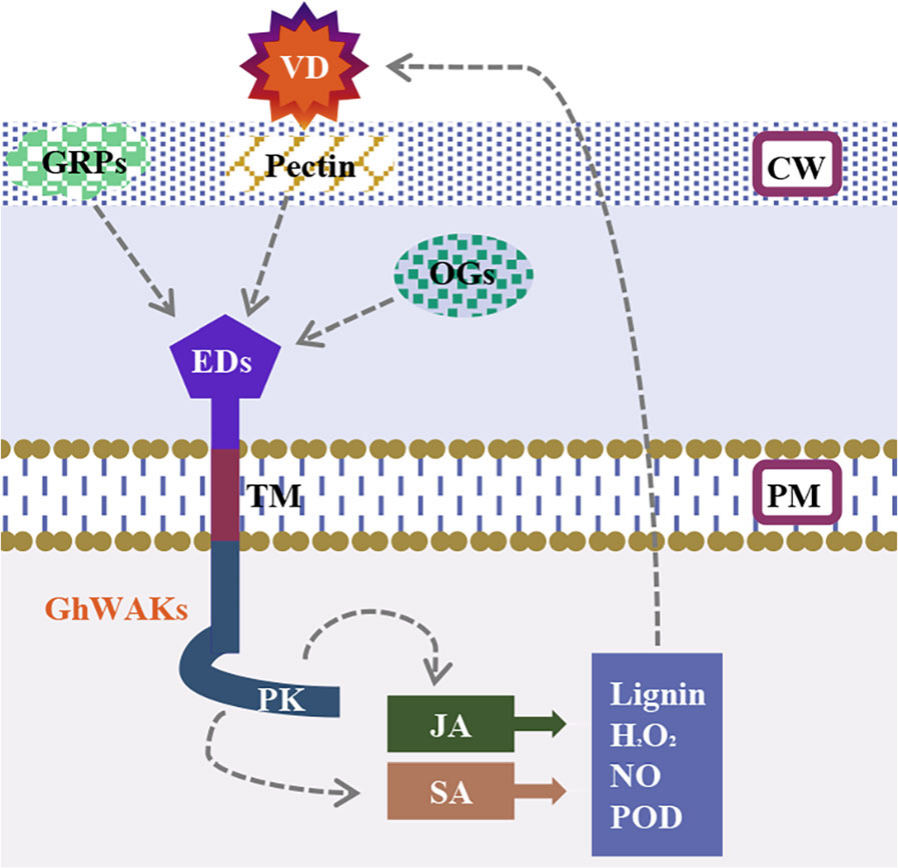
A proposed model explaining how GhWAKs regulate cotton resistance to *V. dahliae*. *V. dahliae* (VD) could secrete various toxins and carbohydrate active enzymes, which break plant cell wall (CW) integrity and generate some degradation products, such as pectin and oligogalacturonides (OGs). GhWAKs, plasma membrane (PM) localizing proteins with transmembrane domain (TM), potentially and directly interact with these cell wall fragments and some cell wall proteins (e.g. glycine-rich proteins, GRPs) by extracellular domains (EDs), and then activate jasmonate (JA) and salicylic acid (SA) signaling pathway via their cytoplasmic pkinase domain (PK). As a result, defence responses are activated, such as the accumulation of lignin, H_2_O_2_ and nitric oxide (NO), and the activity of peroxidase (POD)

In this study, we carried out a genome-wide analysis of WAK gene family in *G. hirsutum* with the identification of 81 putative *GhWAKs*, which might sense different outside signals to trigger intracellular signaling pathways that response to various environment-stresses. Of which, 28 *GhWAKs* with potential roles in the interaction between cotton and *V. dahliae* were screened out. Silencing *GhWAKs* could significantly affect the expression of JA- and SA-related marker genes, increased the spread of *V. dahliae* in the cotton stems, dramatically compromised *V. dahliae*-induced accumulation of lignin, H_2_O_2_ and NO, but enhanced POD activity. These results provided direct evidences that *GhWAKs* participate in the cotton resistance to *V. dahliae*. Finally, a model for how GhWAKs were involved in cotton resistance to *V. dahliae* was proposed (Fig. 9).

## Methods

### Identification and bioinformatics analysis of *GhWAKs*

The amino acid and nucleotide sequences of WAKs from Arabidopsis accessed from TAIR website (https://www.Arabidopsis.org/) were queried against *G. hirsutum* genome database (HAU) in CottonFGD (https://cottonfgd.org/) using BLAST program (E-value < 0.01) [7, 23]. The obtained putative GhWAKs were further identified by HMMER software (HMM Database = Pfam; Significance E-values < 0.01) (https://www.ebi.ac.uk/Tools/hmmer/search/hmmscan) to confirm the presence of conserved protein domains.

Functional sites and transmembrane topology for all putative GhWAKs were analyzed through PROSITE database (https://prosite.expasy.org/) and Phobius database (http://phobius.sbc.su.se/), respectively. The number of amino acids, molecular weight, theoretical isoelectric point and instability index of proteins were analyzed using ExPASy program (http://www.expasy.org/). Prediction of protein subcellular localization was performed using CELLO v2.5 (http://cello.life.nctu.edu.tw/) and ProtComp 9.0 (http://www.softberry.com/berry.phtml?topic=protcomppl&group=programs&subgroup=proloc). Signal peptides were predicted using SignalP 5.0 (http://www.cbs.dtu.dk/services/SignalP/).

### Analysis of chromosomal location, genes structure and *cis*-elements

The information about physical chromosomal locations and gene structures of *GhWAKs* was extracted from the gene annotations in gene feature format (GFF) files, which were downloaded from the CottonFGD website and analyzed by TBtools software [42]. The potential promoter sequences, 2 kb upstream of *GhWAKs*, were also extracted from *G. hirsutum* genome database. The *cis*-elements in the potential promoters were predicted using PlantCARE databases (http://bioinformatics.psb.ugent.be/webtools/plantcare/html/).

### Plant materials and *V. dahliae* inoculation

The seeds of *Nicotiana benthamiana* and *G. hirsutum* cv. Nongda 601 (ND601) were preserved at the State Key Laboratory of North China Crop Improvement and Regulation, Hebei Agricultural University, China. *N. benthamiana* was grown in the greenhouse about 5 weeks at 21 °C with 14/10 h (light/dark) photoperiod. ND601 were grown in the greenhouse at 25 °C under a 14-h light/10-h dark cycle with relative humidity about 70%. Cotton seedlings inoculation with *V. dahliae* strain Linxi 2–1 (10^7^ spores ml^− 1^) was performed as previously described [39].

### Proteins subcellular localization

The ORF of *GhWAK77* (without the stop codon) was amplified by PCR with primers gWAK77-F and gWAK77-R (Table S1), and then introduced into entry vector pDONR™207 by attB/attP recombination reaction, as described by the manufacturer (Invitrogen). The *GhWAK77* fragment was transferred from the entry clone to expression vector pEarlyGate103 [43] with attL/attR recombinant reaction, as described by the manufacturer (Invitrogen). The recombinant expression vector was introduced into *Agrobacterium tumefaciens* GV3101, cultured and infiltrated into four-week-old tobacco leaves via the method described by [44]. After 2 days, GFP signal in the tobacco leaf epidermal cells was examined using a laser scanning microscope (FluoView FV1000; Olympus).

### RNA-seq data and qRT-PCR analysis

The transcription patterns of *GhWAKs* in cotton roots after inoculation with *V. dahliae* were analyzed using high-through RNA-seq data published previously [37]. Log_2_^Fold change^ were calculated from FPKM (fragments per kilobase of exon model per million mapped) and used for the heat map of hierarchical clustering with the TBtools v0.67 software [42]. Total RNA was extracted using EASYspin Plant RNA kit (Aidlab, Beijing, China) according to the manufacturer’s instructions. The quality and concentration of RNA were detected by 1.5% agarose gel electrophoresis and NanoDrop™ 1000 spectrophotometer (Thermo Fisher Scientific), respectively. cDNA was synthesized with a reverse transcription kit (ReverTra Ace® qPCR RT Master Mix with gDNA Remover, TaKaRa, Dalian, China). qRT-PCR was performed using 7500 Real Time PCR System (Applied Biosystems, USA) with THUNDERBIRD®SYBR® qPCR Mix (TaKaRa, Dalian, China). The 2^-*ΔΔCt*^ method was used to calculate the relative expression of genes. *GhHis 3* was used as internal reference. Three biological repeats were taken for each treatment.

### VIGS assays in cotton

The vectors for VIGS, pTRV1 and pTRV2, were kindly provided by Professor Liu Yule of Tsinghua University [45]. The fragments from *GhWAKs* were amplificated by PCR and inserted into the pTRV2 vector between *EcoR* I and *Kpn* I. The constructed vectors were separately transferred into *A. tumefaciens* strain GV3101 by freeze-thaw method [46]. VIGS in cotton was performed as described previously [47]. At least 30 plants were used per treatment, and each treatment was repeated three times. Plant resistance to *V. dahliae* was assayed by analyzing disease index [48].

### Detection and isolation of *V. dahliae* in cotton stems

At 5 dpi and 7 dpi, 1 cm and 0.5 cm of samples excised at a height of 0.5 cm stem above ground were used for detection and isolation of *V. dahliae*, respectively. *V. dahliae* detection by PCR was performed using primers P1 and P2 [49]. *V. dahliae* isolation from cotton stems was carried out according to the previous method [50]. Twenty-four individual plants were sampled for each treatment and repeated three times.

### Measurements of NO, H_2_O_2_ and POD activity

The first true leaves of cotton seedlings were powdered in the mortar with liquid nitrogen and homogenized using 50 mM sodium phosphate buffer (pH 7.0). After centrifugation (14,000 g, 20 min), the supernatants were used for the determination of NO, H_2_O_2_ and POD activity with commercialized assay kits (Nanjing Jiancheng Bioengineering Institute, China), following the manuals. The total protein concentration of the supernatants was measured using Pierce™ BCA Protein Assay Kit (Thermo Scientific).

### Primers and statistical analysis

All primers used in this study were listed in Table S1. Differences between measured values were analyzed using software GraphPad Prism® 8 (GraphPad, San Diego, CA, USA). A two-way ANOVA with multiple comparisons (Sidak’s test) was used to compare gene expression in cotton roots between inoculated with *V. dahliae* and inoculated with water (CK) at the same hpi, disease indices, H_2_O_2_ and NO content, and POD activity between *GhWAK*-silenced plants and CK. A one-way ANOVA with Dennett’s multiple-comparisons test was used to compare lignin content between *GhWAK*-silenced plants and CK. The *P*-value less than 0.05 was assumed to be statistically significant.

## Supplementary Information

**Additional file 1: Table S1**. Primers list.

**Additional file 2: Figure S1**. Gene structures of *GhWAKs*.

**Additional file 3: Figure S2**. Detection of *V. dahliae* in cotton stems by PCR. M, marker. DNA templates were from *V. dahliae* spores as positive control (V), water as negative control (W), and cotton seedling stems (lane 1–4 for 5 dpi and lane 5–8 for 7 dpi).

## Data Availability

All GhWAKs sequence information is available in the Cotton Functional Genomics Database (CottonFGD) (https://cottonfgd.org/about/download.html). The data generated or analyzed during the current study are included in this published article and its supplemental data files and available from the corresponding author on reasonable request.

## Abbreviations

ABA: Abscisic acid
CWI: Cell wall integrity
*EDS*: Enhanced disease susceptibility
EGF: Epidermal growth factor
ET: Ethylene
FPKM: Fragments per kilobase of exon model per million mapped
GA: Gibberellin
hpi: Hours post inoculation
*ICS*: Isochorismate synthase
*JAZ*: Jasmonate-zim-domain protein
*LOX*: Lipoxygenase
MeJA: Methyl jasmonate
NO: Nitric oxide
*NPR*: Nonexpresser of PR protein
PM: Plasma membrane
POD: Peroxidase
PR: Pathogenesis related protein
RLKs: Receptor-like kinases
ROS: Reactive oxygen species
SA: Salicylic acid
SP: Signal peptide
TM: Transmembrane
VIGS: Virus-induced gene silencing
WAKs: Wall-associated kinases
