# Supplementary material for: Identification of cell wall-associated kinases as important regulators involved in Gossypium hirsutum resistance to Verticillium dahliae
Source: BMC Plant Biol. 2021 May 15;21:220. doi: 10.1186/s12870-021-02992-w (PMC8122570; doi:10.1186/s12870-021-02992-w)
Supplement: Supplementary file 1 — Additional file 1: Table S1. Primers list. [file 12870_2021_2992_MOESM1_ESM.docx]

**Table S1. Primers list**

| **Primer name** | **Primer sequence (5' to 3')** | **Use and specification** |
| --- | --- | --- |
| gWAK77-F | GGGGACAAGTTTGTACAAAAAAGCAGGCTTCATGATGTCGTGCTATGCAA | For subcellular localization |
| gWAK77-R | GGGGACCACTTTGTACAAGAAAGCTGGGTGTTCTCGCCGAGATGAATTA |  |
| GhHis3-F | TCAAGACTGATTTGCGTTTCCA | For RT-qPCR analysis |
| GhHis3-R | GCGCAAAGGTTGGTGTCTTC | For RT-qPCR analysis |
| GhWAK1-RT-F | TCAACGGATTTGGGTCGGTA | For RT-qPCR analysis |
| GhWAK1-RT-R | TGGTGGAATTGACGTGATGC | For RT-qPCR analysis |
| GhWAK4&45-RT-F | CAGTTGCGCATCCTTTCTCA | For RT-qPCR analysis |
| GhWAK4&45-RT-R | ACGTTGTCCGTAGGAACCTT | For RT-qPCR analysis |
| GhWAK5&49-RT-F | CAAACTCCAGCTGCCAAAGT | For RT-qPCR analysis |
| GhWAK5&49-RT-R | TTTGCTGGGTGTGAAACTGG | For RT-qPCR analysis |
| GhWAK10&55-RT-F | AGCAGAAGAGCTCGAACGTA | For RT-qPCR analysis |
| GhWAK10&55-RT-R | CGATTCCCGATGATCAGTGC | For RT-qPCR analysis |
| GhWAK26-RT-F | GACTGCTTTGATCGCAGGTT | For RT-qPCR analysis |
| GhWAK26-RT-R | TTGTTTCACGTTCCTTGCGT | For RT-qPCR analysis |
| GhWAK31&77-RT-F | TCCACAGCGATAACTGGGTT | For RT-qPCR analysis |
| GhWAK31&77-RT-F | TCCTCCATGCAGCAACCTAA | For RT-qPCR analysis |
| GhWAK48-RT-F | TCAAACTCCTCGGATGCTGT | For RT-qPCR analysis |
| GhWAK48-RT-R | GCTGTTTCAGTGGCGATTCT | For RT-qPCR analysis |
| GhWAK69-RT-F | CTGTTGCTTGGAGACCGAAG | For RT-qPCR analysis |
| GhWAK69-RT-R | GGATGGATGCAGATGAGTGC | For RT-qPCR analysis |
| GhWAK72-RT-F | AAACCGGACTGCTTTGATCG | For RT-qPCR analysis |
| GhWAK72-RT-R | TTGTTTCACGTTCCTTGCGT | For RT-qPCR analysis |
| GhWAK26/72-V-F | GAATTCGCTTTGATCGCAGGTTTAAC | For VIGS vector construction |
| GhWAK26/72-V-R | GGTACCACATTTGACAGCAACCGGAG | For VIGS vector construction |
| GhWAK31/77-V-F | GAATTCCATAACTTCCAAGGACATCATC | For VIGS vector construction |
| GhWAK31/77-V-R | GGTACCGAAAACAAGAAACGGCACTG | For VIGS vector construction |
| P1 | CATCAGTCTCTCTGTTTATACCAACG | For *V. dahliae* detection |
| P2 | CGATGCGAGCTGTAACTACTACGCAA | For *V. dahliae* detection |

Note: Restriction sites are underlined.
