# Supplementary figures and images for: Identification of cell wall-associated kinases as important regulators involved in Gossypium hirsutum resistance to Verticillium dahliae
Source: BMC Plant Biol. 2021 May 15;21:220. doi: 10.1186/s12870-021-02992-w (PMC8122570; doi:10.1186/s12870-021-02992-w)

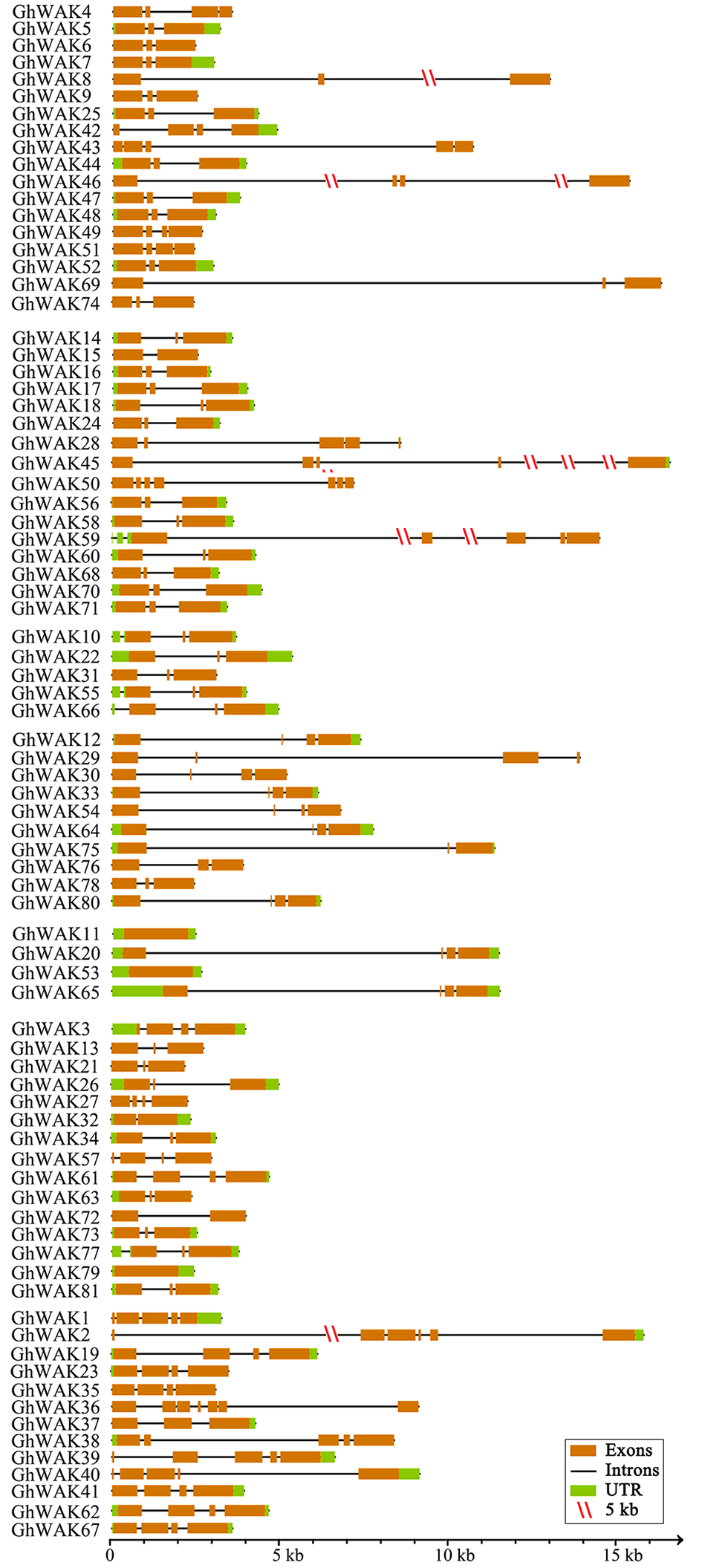

Supplement: Supplementary file 2 — Additional file 2: Figure S1. Gene structures of GhWAKs. [file 12870_2021_2992_MOESM2_ESM.tif]

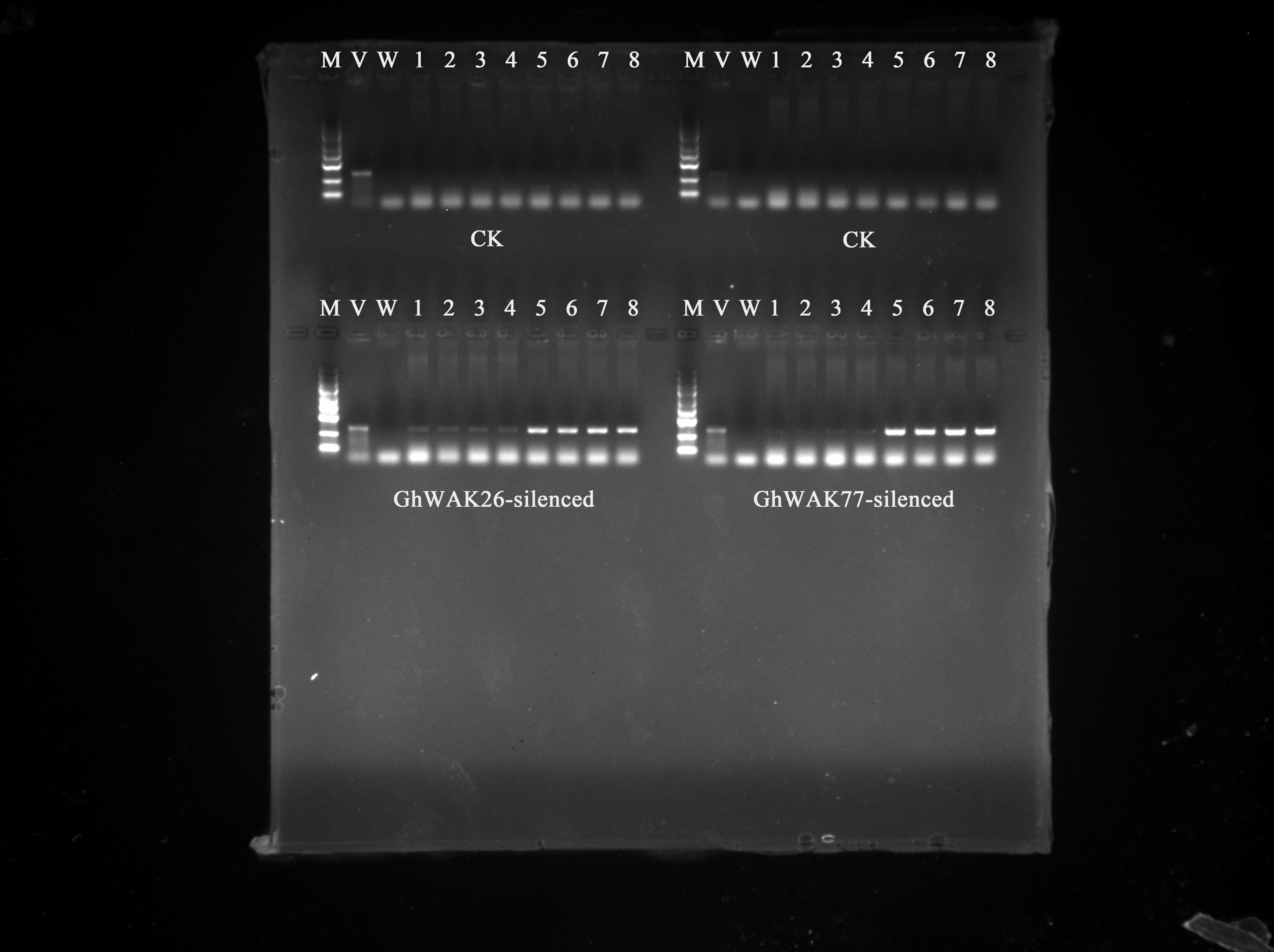

Supplement: Supplementary file 3 — Additional file 3: Figure S2. Detection of V. dahliae in cotton stems by PCR. M, marker. DNA templates were from V. dahliae spores as positive control (V), water as negative control (W), and cotton seedling stems (lane 1–4 for 5 dpi and lane 5–8 for 7 dpi). [file 12870_2021_2992_MOESM3_ESM.tif]
